# Supplementary material for: Immune and Reproductive Biomarkers in Female Sea Urchins Paracentrotus lividus under Heat Stress
Source: Biomolecules. 2023 Aug 4;13(8):1216. doi: 10.3390/biom13081216 (PMC10452167; doi:10.3390/biom13081216)
Supplement: Supplementary file 1 [file biomolecules-13-01216-s001.zip › biomolecules-2507733-supplementary.pdf]

Supplementary material

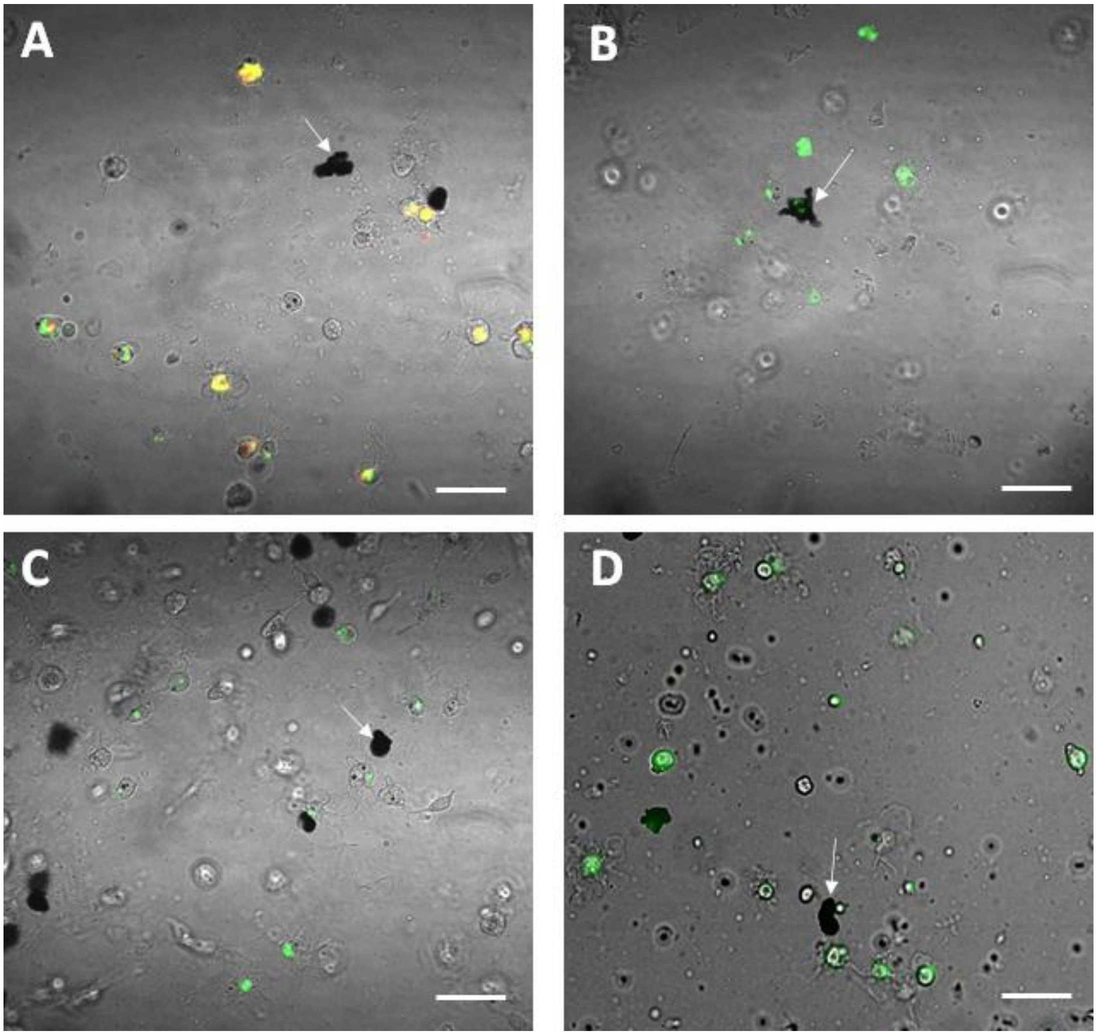

Figure S1. Representative images of *P. lividus* coelomocytes analyzed by laser confocal microscope for mitochondrial membrane potential assessed by JC-1 staining (A), intracellular pH assessed by BCECF-AM staining (B), H_2O_2 content assessed by H_2DCFDA staining (C), and reactive nitrogen species (RNS) content assessed by DAF-DA staining (D). White arrows indicate red amoebocytes. Bar = 20 μm
